# The Role of Pulmonary Drug Delivery in Modern Therapeutics: An Overview

**DOI:** 10.7759/cureus.68639

**Published:** 2024-09-04

**Authors:** Prem Kumar Subramani, Remya P N, Damodharan Narayanasamy

**Affiliations:** 1 Pharmacy, SRM College of Pharmacy, SRM Institute of Science and Technology, Chennai, IND; 2 Pharmacy, SRM Institute of Science and Technology, Chennai, IND; 3 Pharmaceutics, SRM Institute of Science and Technology, Chennai, IND

**Keywords:** formulation of drugs, devices for pulmonary disease conditions, approaches in pulmonary drug delivery, drug deposition, lung infection

## Abstract

The pulmonary drug delivery system is a promising and evolving technology in which the prescribed medicine is breathed through the lungs, and subsequently, it enters the circulation via the alveolar epithelium. This category of pulmonary drug delivery system is an appealing and non-invasive administration method. Pulmonary drug delivery is most commonly utilized to treat airway problems by providing locally active medicines directly to their site of action. The dose required to have a pharmacological effect is reduced when medicines are delivered directly to their site of action. In addition to locally acting medications, the pulmonary route can be utilized to deliver compounds with systemic effects, such as in the case of insulin inhalation therapy for systemic absorption. Particle size, bioavailability, device compatibility, and other aspects must be addressed, including the formulation of drugs into an acceptable dosage for inhalation with sufficient stability. This formulation must also be used in conjunction with a suitable inhaler device that produces an aerosol with a particle or droplet size that assures deposition in the required targeted area of the pulmonary system. Recent advancements in pulmonary drug delivery include the development of targeted nanoparticles and inhalable biologics, which enhance drug absorption and efficacy while minimizing systemic side effects. Future directions focus on personalized medicine approaches and advanced inhalation technologies, although limitations such as variable patient adherence and the need for precise dosing continue to pose challenges.

## Introduction and background

Lung disorders, such as asthma and chronic obstructive pulmonary disease (COPD), affect a significant portion of the global population, with asthma impacting over 300 million people globally, especially in developed countries, while COPD affects over 380 million, particularly in low- and middle-income regions, making it the third leading cause of death worldwide. Lung cancer, the second most common type of cancer, and these respiratory conditions are expected to rise due to aging populations and environmental factors. Despite the availability of various treatment options, these disorders continue to pose substantial challenges in clinical management. Traditional systemic drug delivery methods, where medications are administered orally or intravenously, often result in limited efficacy for lung diseases due to poor drug targeting and increased systemic side effects. This limitation has driven the exploration of alternative delivery routes, with the pulmonary route emerging as a particularly effective approach.

The effectiveness of a drug is primarily dependent on its inherent properties and the specific disease condition it is used for. Targeting the medication directly to the lungs through inhalation allows for a localized approach, which can reduce the need for higher doses and lower overall medication concentrations in the body. This localized approach helps minimize potential negative side effects of the drug, as the treatment is focused on the specific area where it is needed. In addition to the corticosteroids, antibiotics, and mucolytics currently available, many medications are being investigated for direct administration to the lungs. Clinical trials on lung cancer patients demonstrate the limited efficacy of systemic medication administration, which may be due to insufficient drug delivery to the tumor. For instance, systemic chemotherapy often fails to deliver adequate drug concentrations to the lung tumors, leading to suboptimal treatment outcomes. Aerosolized chemotherapy could maximize exposure while minimizing negative effects, further highlighting the advantages of targeted pulmonary treatment. Pulmonary chemotherapy represents another approach to localized drug delivery, particularly for the treatment of lung cancer. Chemotherapeutic agents such as 5-fluorouracil and doxorubicin have been administered via inhalation, allowing direct targeting of the tumor site within the lungs.

Numerous studies have demonstrated that this method can achieve significant therapeutic responses, with the potential to enhance drug efficacy while minimizing systemic toxicity. In addition, inhalation-based delivery of these agents has been associated with a reduction in fibrosis and other pulmonary complications, highlighting its potential advantages over traditional systemic chemotherapy. However, despite these promising results, pulmonary chemotherapy remains an experimental approach, and its clinical success is yet to be fully established. Another innovative application of pulmonary drug delivery is in the administration of mucosal vaccines, which target the primary entry sites of infections. There is a limited number of mucosal vaccines, including Convidecia-Air (Ad5-nCoV), an orally inhaled vaccine approved in China, and iNCOVACC (BBV154), along with Flumist, an influenza vaccine that remains commercially available. These vaccines represent a crucial advancement in immunization strategies, particularly given that approximately 90% of pathogens enter the body through the oral or nasal route. By directly stimulating immune responses in the respiratory tract, these vaccines can potentially offer superior protection compared to systemic vaccines. Moreover, mucosal vaccines may also reduce the transmission of infectious agents by targeting the mucosal surfaces where many pathogens first establish infection, thereby interrupting disease spread at its source. This approach underscores the growing interest in developing vaccines that not only prevent disease but also interrupt transmission at its source.

The problem that this review addresses is the ongoing challenge of effectively delivering drugs to the lungs, particularly in the treatment of chronic and life-threatening lung conditions. Despite advancements in pulmonary drug delivery, there remains a need to explore and optimize these systems for better clinical outcomes [1,2]. The objective of this text is to provide a comprehensive overview of pulmonary drug delivery systems, including the mechanisms of drug deposition in the lungs, the types of formulations and devices used, and the advantages of targeted drug delivery for treating pulmonary diseases. By examining the current state of research and clinical applications, this review aims to highlight the potential of pulmonary drug delivery to improve therapeutic outcomes for patients with lung disorders.

## Review

Mechanism of drug deposition in lungs

In the past few decades, numerous medications have demonstrated systemic absorption following pulmonary delivery in both animals and humans. Pulmonary delivery is achieved through intranasal or oral inhalation routes. Irrespective of the delivery method, the efficiency of nasal delivery can vary based on the device used, drug formulation, and the subject's inhalation technique. Oral inhalation of drugs is commonly referred to as intra-tracheal instillation or intra-tracheal inhalation. In laboratories, intravenous injection is the most common technique. Trans-tracheal administration involves injecting a small quantity of medication solution or dispersion directly into the lung through an intra-tracheal syringe. This method is efficient and allows for measurable drug delivery to the lungs. The effectiveness of the drug is mainly dependent on the particle size and characteristics of the aerosol particles, as well as the specific region of the lung that is targeted. In preclinical animal studies, intra-tracheal instillation is frequently used to evaluate pulmonary absorption and systemic bioavailability, particularly concerning precise dosage and efficacy. However, the findings from these studies may not directly translate to aerosol applications in humans, where the inhalation technique enables more uniform dispersion and deeper lung penetration [3]. Although more expensive, this approach makes it more challenging to determine the exact dosage in the lungs.

Three processes are responsible for the deposition of medication in the pulmonary airways following aerosol administration: gravity sedimentation, inertial impaction, and diffusion.

If the particle size of the medication is larger, either hyperventilation or gravitational force will cause deposition. Smaller particles frequently deposit through a process called Brownian motion, which is based on diffusion. In addition to pulmonary morphology and ventilator characteristics, other crucial factors to consider include particle or droplet size and shape. When fibers are present, their hygroscopic properties and form significantly influence the extent of medication deposition during inhalation. For instance, the mass median aerodynamic diameter (MMAD), based on the physical characteristics of the particulate system, is used.

**Figure 1:**
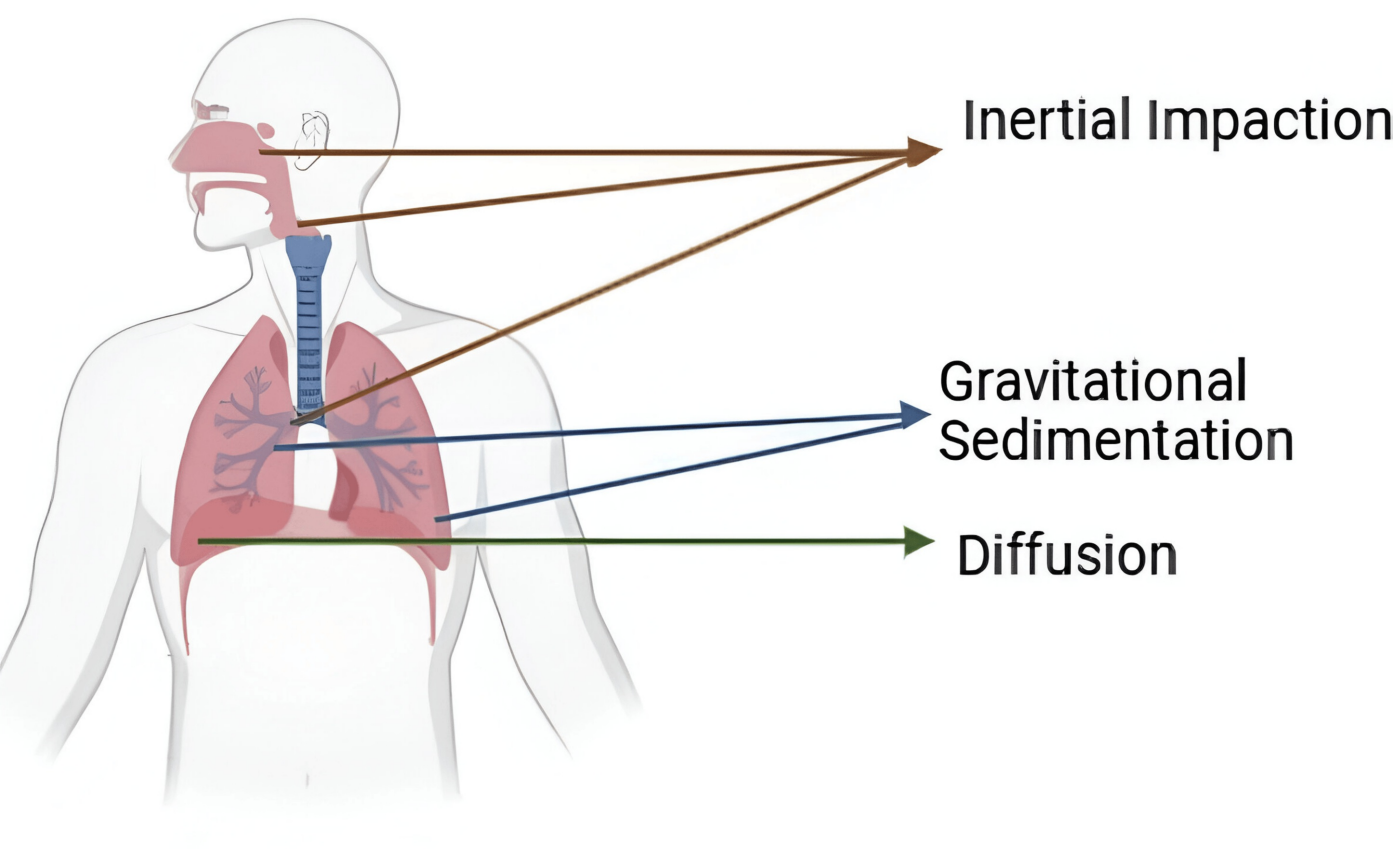
Mechanism of drug deposition. The respective image is the original work of the author representing the mechanism of drug deposition [2,3].

**Figure 2:**
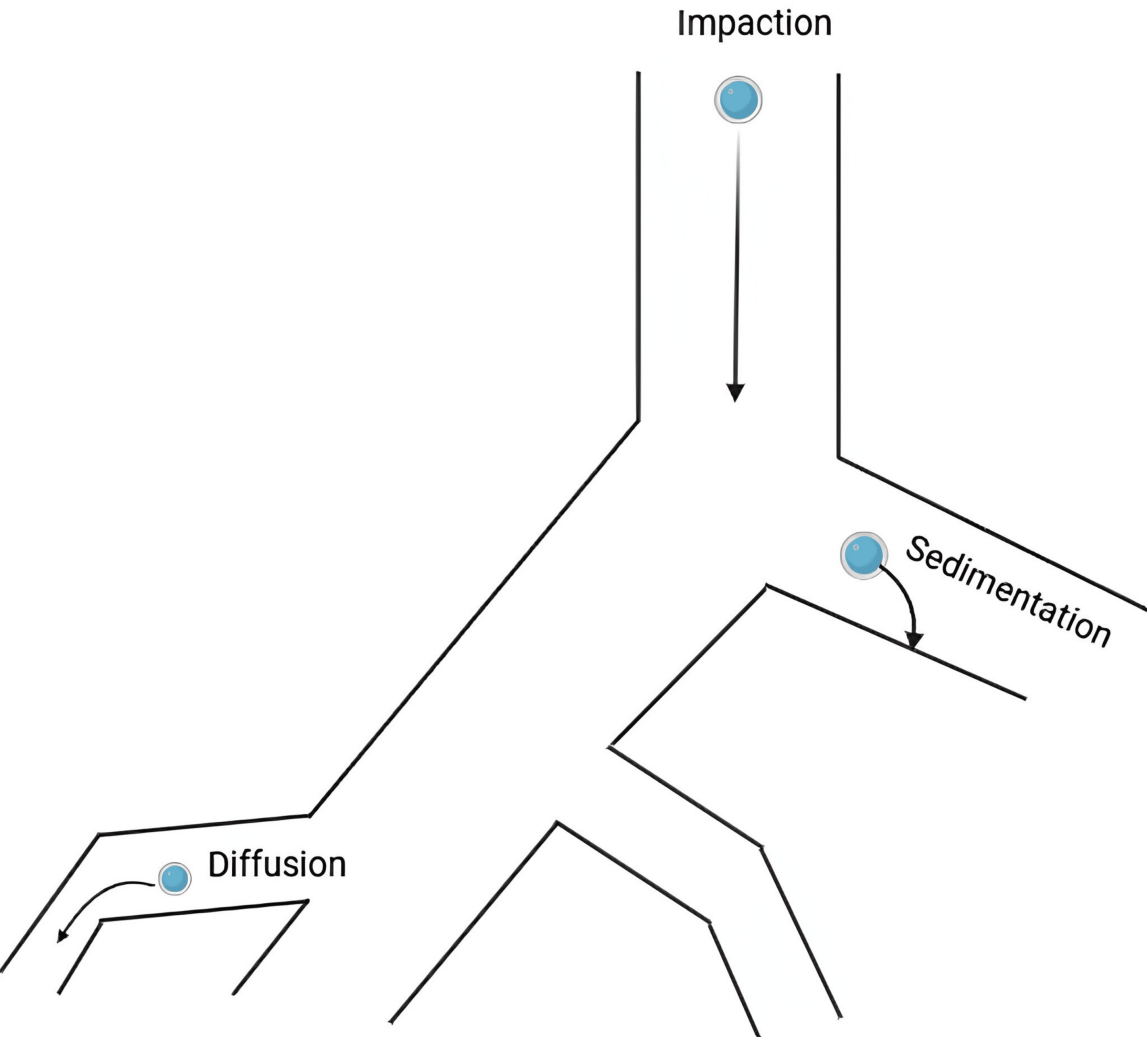
Schematic representation mechanism of drug deposition The respective image is the original work of the author representing the schematic mechanism of drug deposition [2,3].

Figure 1 and Figure 2 illustrate the drug deposition mechanism influencing the deposition of inhaled particles in the respiratory system. Inertial impaction occurs in the upper airways, such as the nasal cavity and throat, where larger particles cannot navigate bends and bifurcations due to their inertia, leading to deposition on airway walls. Gravitational sedimentation takes place in the smaller bronchioles, where airflow velocity decreases, causing medium-sized particles to settle out of the airstream and deposit on airway surfaces. Diffusion, relevant for very small particles like nanoparticles, involves random movement due to Brownian motion, allowing these particles to reach the deeper regions of the lungs, including the alveoli. Each of these mechanisms is influenced by factors such as particle size, shape, and density, as well as airflow dynamics. Understanding these processes is crucial for optimizing drug delivery via inhalation, ensuring that medication effectively reaches targeted regions of the lungs.

Approaches in pulmonary drug delivery

The most important and vital aspect of medication delivery methods is lung-targeted drug delivery systems. This approach is particularly effective because it allows for localized therapy within the lungs, enabling site-specific drug delivery at higher concentrations. As a result, pulmonary drug delivery can achieve therapeutic effects with very small doses compared to systemic administration. However, the actual percentage of the original dosage delivered to the lungs can vary significantly based on factors such as drug formulation, particle size, and delivery method, meaning that the typical estimate of 10-20% of the original dosage may not apply uniformly across all medications. This method enhances local drug activity while significantly reducing systemic side effects. Researchers have introduced innovative features in drug delivery systems via the pulmonary route. For instance, lactose-drug combinations are used in dry powder inhalers (DPI) for immediate release, while nanoparticles are employed for controlled release. In addition, liposomes, micelles, and microparticles are utilized, often relying on polymers to regulate the release of the medication.

Microspheres

The use of aerosolized microspheres via the pulmonary route often allows for the controlled release of medication, which can be effective for both respiratory and non-respiratory diseases. The characteristics of these microspheres, including their morphological shape, size, and porosity, are critical factors that influence their formulation and performance during the preparation process. While many microspheres are small and designed to minimize moisture absorption, their behavior in the presence of moisture can vary depending on the specific formulation and environmental conditions in the lungs [4].

Nanoparticles

Nanoparticles are primarily composed of polymers, with the drug either entrapped in their core or bound to their surface. This unique structure provides protection against enzymatic degradation, enhances bioavailability, and facilitates controlled release of the medication. Recent advancements in drug delivery through nanotechnology have demonstrated numerous advantages, such as improved treatment efficacy and a reduced risk of systemic side effects. By harnessing the capabilities of nanoparticles for targeting lung disorders, researchers have made significant strides in drug delivery, addressing various challenges in the field. This innovative approach has effectively improved the bioavailability and pharmacokinetics of medications, paving the way for enhanced treatment outcomes in patients with lung disorders [5].

Liposomes

Liposomal drug formulations for aerosol delivery offer several significant benefits and applications, including aqueous compatibility, sustained pulmonary release, and the facilitation of intracellular delivery to specific alveolar macrophages, which helps prevent local irritation and reduces toxicity. Liposomal drug formulations can be used both locally and systemically, providing the distinct advantage of improved efficacy with reduced toxicity. While liposomes are a promising drug delivery system with high drug entrapment efficiency, they are particularly effective for lipophilic drugs. Hydrophilic drugs tend to be poorly encapsulated in liposomes, which can lead to leakage and reduced effectiveness.

Micelles

Micelles are spherical aggregates formed when amphiphilic surfactant molecules arrange themselves in solution, with hydrophilic heads facing outward and hydrophobic tails oriented inward. This structure allows micelles to encapsulate hydrophobic substances within their core, facilitating the solubilization and transport of poorly water-soluble drugs. A hydrophilic shell surrounds nanoparticles, protecting the encapsulated drug and potentially preventing recognition by the reticuloendothelial system [6].

Cyclodextrins

Cyclic oligosaccharides, such as cyclodextrins (CDs), are produced as a result of oligosaccharide association. They contain six, seven, or eight glucopyranose units, referred to as α-, β-, or γ-CD, respectively. These cyclodextrins frequently form non-covalent bonds, making them highly soluble in liquid media. Cyclodextrins are commonly utilized in pharmaceutical research due to their suitable size, favorable drug-molecule interactions, and low manufacturing costs. Research has investigated the potential for CDs to deliver pharmaceuticals directly to the lungs. Both testosterone and salbutamol can be encapsulated within CDs. They particularly improve the solubility and bioavailability of poorly water-soluble drugs. These formulations enhance drug stability and facilitate controlled release, leading to improved therapeutic effects in respiratory diseases such as asthma and chronic obstructive pulmonary disease (COPD). Clinical studies have demonstrated that cyclodextrins can effectively reduce the required doses of certain medications while minimizing side effects, resulting in better patient compliance and overall treatment outcomes. In addition, the potential for encapsulating a wide range of therapeutics, including peptides and proteins, positions cyclodextrin formulations as versatile options for targeted pulmonary therapy.

Devices

DPIs

They are categorized as passive devices, which are commonly used, and active devices. Both devices are distinguished based on powder aerosolization. The performance of DPIs depends on the powder formulation and the design of the inhaler device [7]. Furthermore, they are subdivided into three categories, namely, single-dose reusable, multi-dose, and single-use devices.

Passive Devices

The most common type of DPIs available on the market is passive devices. In this method, the patient's inhalation provides the energy needed to aerosolize the medication, which is in powder form. These passive devices consist of an air input, a dispersion chamber, and a mouthpiece. In conventional passive inhalers, the airflow within the dispersion chamber disperses the medication powder into inhalable aerosols, allowing the medicine to be inhaled. Traditional passive inhalers use dispersion mechanisms where drug powder is dispersed into inhalable aerosols when the equilibrium between cohesive forces and de-agglomeration forces, created by the airflow, is achieved. The advantages of DPIs are leveraging the patient's inhalation to aerosolize medication, eliminating the need for external energy sources, and simplifying the design. They are generally lightweight and easy to use, promoting portability for patients. In terms of the disadvantages, the effectiveness of passive DPIs can vary based on the patient’s inhalation effort, which may lead to inconsistent dosing and reduced efficacy, especially in individuals with compromised pulmonary function.

Active Devices

Active DPI devices are used as multi-dose DPIs due to their independent aerosolization efficiency. In these devices, the drug powder is dispersed by external energy sources, such as compressed air or electrical vibration. Active multi-dose DPI devices can achieve satisfactory fine particle fraction (FPF) values, typically in the range of 50-70%. These devices are utilized in various pharmaceuticals, including spray-dried insulin. The advantage of active devices is their consistent aerosolization performance, which is not dependent on patient inhalation effort. However, this characteristic can be a disadvantage for patients with reduced pulmonary function. Micro-dose DPIs often use compressed air and piezoelectric systems for dispersion [8]. In terms of advantages, active DPIs provide consistent aerosolization performance through external energy sources, ensuring reliable drug delivery regardless of the patient's inhalation effort. This results in satisfactory fine particle fraction (FPF) values, enhancing medication deposition in the lungs. In terms of disadvantages, the reliance on external energy sources may increase the complexity and cost of these devices. In addition, they may not be suitable for patients with limited pulmonary function, as the required energy for effective aerosolization can be higher than what they can generate.

Multi-dose Device

The NEXThaler® is a multi-dose inhaler designed with a simple technique. The dose is administered by opening the lid and inhaling the medication through the mouthpiece, typically within a minute. After taking the dose, the lid must be closed. This inhaler features a novel breath-actuated mechanism and an innovative full-dose feedback system to ensure the counter is activated only once the entire dose has been released. One of the key benefits of the NEXThaler® is its effective protection against light and moisture, as well as its provision of an additional pack for each dose unit. The inhaler includes a dosage counter to indicate the remaining doses. The main advantage of this multi-dose device is its ability to offer robust protection against light and moisture, along with the convenience of an extra pack for each dose unit. It is essential to have either a mechanical or electrical dose counter to display the remaining dose. The advantage of the NEXThaler® is that it features a user-friendly design with a breath-actuated mechanism and a dosage counter, promoting accurate dosing and adherence to treatment. Its effective protection against light and moisture also ensures drug stability, contributing to its reliability. Despite its advantages, the complexity of the design may pose a learning curve for some patients, and the effectiveness of the counter relies on proper functioning, which may be a concern if it malfunctions.

Single-Dose Reusable Device

Single-dose reusable devices utilize individual units, such as capsules or blisters, for dose preparation. After the patient uses the inhaler to aerosolize the single dose, the unit is discarded. The design of these devices has minimized dimensions, making them compact. The powder mass can be adjusted within each unit, accommodating both lower and higher-dose therapies. Single-dose reusable devices are often preferred over multi-dose inhalers for administering high-dose medications due to their efficiency and precision. In terms of advantages, single-dose reusable devices are compact and designed for precision in delivering high-dose medications, making them efficient for therapies that require accurate dosing. Their use of individual units ensures that each dose is fresh and uncontaminated. In terms of disadvantages, the need for individual unit preparation can be inconvenient for patients, and the disposal of used units may contribute to environmental waste. In addition, the design may limit the volume of medication that can be delivered in a single dose.

Single-Use Inhalers

Single-use inhalers are well-suited for infrequent therapies or specific infectious diseases. For instance, the TwinCaps® DPI by Hovione is a classic example of a non-reusable device designed for single doses of 40 mg. It has been used to deliver powdered aerosols of Laninamivir (Inavir®), a long-acting neuraminidase inhibitor for influenza treatment. Single-use inhalers help reduce the risk of cross-infection and are effective for vaccine delivery through inhalation. Chronic infections in cystic fibrosis (CF) patients are treated with the antibiotic Colistin, delivered using non-reusable devices like the Twincer®. This device is engineered with advanced air equipment, offering high aerosol performance (with a fine particle fraction [FPF] ranging from 58% to 67%) and is compatible with lactose-based formulations. The cost-effectiveness and ease of use of single-use inhalers are enhanced by their design. The Twincer® comprises three plastic parts, while the TwinCaps® has two, simplifying construction and reducing expenses. In addition, the straightforward process protocol encourages adherence to irregular use patterns with these non-reusable devices [9]. In terms of advantages, single-use inhalers are ideal for infrequent therapies and specific conditions, minimizing the risk of cross-infection and ensuring hygienic delivery of medications. They are straightforward to use, encouraging adherence, especially in patients with irregular usage patterns. In terms of disadvantages, the disposable nature of these devices may lead to increased waste and cost over time. Furthermore, they may not be suitable for patients requiring frequent dosing or long-term therapies due to limited medication capacity.

Pressurized Metered-Dose Inhalers

Pressurized metered-dose inhalers (pMDIs) are sometimes considered outdated devices, with relatively few advancements over the past 50 years. However, new compact and user-friendly designs, such as breath-actuated, breath-responsive, and velocity-adjusted devices, have addressed issues related to actuation direction, limited unit range, and incomplete dose delivery. Despite facing strong competition from DPIs and novel soft-mist inhalers, the revitalized pMDI remains significant due to its affordability, which is especially important in developing markets. Pressurized metered-dose inhalers can be challenging to use, especially for children and the elderly, as they require precise coordination between pressing the canister and inhaling. If not shaken properly, the medication may not mix evenly, leading to inconsistent doses. Some users may also experience throat irritation or coughing due to sensitivity to the propellant. The cold sensation from the spray can be uncomfortable for some, and although most pMDIs now use eco-friendly propellants, there are still concerns about the environmental impact of older CFC-based inhalers. Key areas of innovation in pMDI technology include dose counters, breath-actuated devices, and electronic adherence measures. Some pMDI hardware technologies are discussed in detail by Stein. For advantages, pMDIs are cost-effective and widely available, making them accessible, especially in developing markets. Recent innovations, such as breath-actuated models and dose counters, have improved their usability and dosing accuracy. For disadvantages, traditional pMDIs may have challenges with dose delivery consistency, particularly for patients with coordination difficulties. The reliance on pressurized systems can also lead to concerns about the environmental impact of propellants.

Dose Counters

The inadequacies of dose counters in some newer pMDIs were highlighted by the US FDA in 2003. Measuring doses by eye using an opaque aluminum canister can be challenging, as patients often struggle to determine when the pMDI is empty. Consequently, dose counters are now considered the most accurate and reliable method for tracking the remaining doses. Without a counter, pMDIs can be used beyond their recommended dose range, potentially leading to suboptimal treatment, which can be hazardous. Dose counters typically rely on various mechanisms, such as sound, pressure, temperature, and canister movement or thumb pressure. However, the effectiveness of these counters can be variable, as their performance may not always correlate directly with the actual delivery of the dose. Currently, products featuring integrated dose counters include ProAir® HFA (Teva Pharmaceutical Industries) and Ventolin® HFA [10].

Breath-Actuated Inhalers (BAIs)

Breath-actuated pressurized metered-dose inhalers (pMDIs) have the potential to address a common issue in traditional pMDI systems: the need for coordinated actuation. Compared to other devices, numerous studies have shown that breath-actuated inhalers (BAIs) can improve outcomes for asthma and lung function. Newer models have continued to evolve. The first breath-actuated pMDI, introduced by 3M Pharma in the mid-1970s (Auto-Haler), has been followed by more advanced devices. The K-Haler™ (Clinical Designs Ltd.) is a recent example, offering improvements in ease of use and functionality. The K-Haler™ features a novel K-Valve™ with kinked-hose technology. When the mouthpiece cap is fully opened, the hose of the K-Valve™ remains kinked, similar to a kink in a hosepipe, keeping the valve closed. When the canister stem is depressed, the device is actuated, allowing a single dose of aerosolized medication to enter the K-Valve™. The device is primed, and because the hose remains kinked, the medication is held in the valve. During inhalation, the tube straightens, releasing the dose. An optional dose counter, such as the Helix™ (Clinical Designs Ltd.), can also be integrated into the design. Bell and Newman provide a detailed explanation of mature breath-actuated pMDI systems. Breath-actuated inhalers work by using the patient's breath to trigger the release of the medication. When the user inhales through the mouthpiece, the inhaler detects the airflow and automatically releases a dose of medication. This mechanism eliminates the need for hand-breath coordination, as the inhaler only activates when the patient inhales with sufficient force. BAIs typically employ either a spring-loaded mechanism or a valve that opens when the inhalation flow reaches a certain threshold, ensuring that the drug is delivered directly to the lungs at the right moment [11]. In terms of advantages, BAIs enhance patient compliance by eliminating the need for coordinated actuation, making them user-friendly for those with respiratory issues. They have been shown to improve medication delivery and lung function outcomes compared to traditional inhalers. Although BAIs are easier to use, they can be more expensive than standard pMDIs. Patients may also face a learning curve with newer technologies, potentially impacting their initial usage effectiveness.

Nebulizers

Nebulizers are especially beneficial for diseases requiring high doses, such as cystic fibrosis, and for individuals who cannot coordinate or achieve the necessary flow rates with other inhalation devices, such as young children. They are classified into three main types based on how they convert medication solutions or suspensions into inhalable aerosols. Jet nebulizers, like the "PARI LC SPRINT®," use propellant gas to disperse liquid medicine into aerosol particles. Jet nebulizers are versatile and cost-effective devices that are easy to use and can deliver larger doses of medication using various breathing gases. However, they can be noisy, take longer to administer medication, are bulkier, may waste some medication, and usually require a power source. Ultrasonic nebulizers, such as the PolyGreen® KN-9210, are quieter and more portable but are less suitable for thermally unstable medications, like proteins, because of the heat generated in the liquid reservoir. Ultrasonic nebulizers are quiet, efficient, and portable devices that produce fine aerosol droplets for effective medication delivery. However, they can be more expensive, may require specific medications, and can potentially alter the properties of some drugs due to heat generated during the nebulization process. Vibrating mesh nebulizers represent the latest technology, with devices such as the Pari eFlow® Rapid offering quick treatment times, minimal residual volume, and improved aerosol distribution. However, their high cost limits their use. Aztreonam is approved for nebulization with the Pari eFlow® mesh nebulizer to treat chronic *P. aeruginosa *airway infections [12]. Vibrating mesh nebulizers are compact, efficient, and produce a consistent aerosol particle size, ensuring effective drug delivery with minimal waste. However, they can be more expensive than other nebulizer types and may require more maintenance to prevent clogging of the mesh. Nebulizers are suitable for delivering high doses of medication and are especially beneficial for patients unable to use other inhalation devices, such as young children or those with severe respiratory issues. They can effectively aerosolize a wide range of medications, providing flexibility in treatment options. However, jet nebulizers can be bulky and noisy, leading to longer treatment times and discomfort. Ultrasonic nebulizers may generate heat that can damage sensitive medications, while vibrating mesh nebulizers, though advanced, can be costly and less accessible for some patients.

Formulation

Types of Formulations Based on Compounds

Formulations for wet aerosol: In the treatment of lung infections in both children and adults, nebulizers frequently produce liquid antibiotic aerosols through mechanical or electrical means. New nebulizer designs have been evaluated, revealing that traditional nebulizers are often bulky and inefficient. In contrast, vibrating mesh nebulizers offer minimal residual volume, fast production, and improved drug delivery efficiency, despite their higher cost. This category also includes surface acoustic wave (SAW) microfluidic atomization, which has demonstrated higher delivery efficiency with a fine particle fraction. Vibrating mesh nebulizers are advanced devices that produce a fine mist of aerosolized medication through a vibrating mesh, ensuring efficient and consistent drug delivery. In a pediatric patient with cystic fibrosis, these nebulizers offer significant benefits over traditional models by being compact and quiet and reducing medication wastage. For instance, using a vibrating mesh nebulizer for tobramycin administration ensures nearly all of the drug reaches the lungs, improving treatment outcomes. This novel technique avoids damage to shear-sensitive natural biomolecules, such as antibodies and plasmid DNA, thereby reducing drug loss associated with high-shear nebulization. Modern nebulization systems can also leverage digital software for process control and performance feedback to optimize medication delivery.

Formulation of DPIs: Devices that use DPIs are typically compact and portable, with short treatment durations that contribute to better patient adherence. In addition, dry powder mixtures of medicines and additives can be more chemically stable and less likely to be contaminated by microbes than liquid formulations, but this depends on the specific formulation and storage conditions. Examples of effective DPIs include colistin methanesulfonate and tobramycin, which are noted for their improved treatment efficiency and patient adherence. However, some powder formulations may become unstable in conditions of excessive humidity. Unlike other inhalers, DPIs do not require propellants. These small devices enable patients to deliver the medication formulation effortlessly [13].

Compounds of antibiotics in combination: Antibiotic monotherapy can contribute to the development of resistance in some cases, which is why antibiotic combinations are often used in clinical settings to reduce this risk, broaden the spectrum, or achieve synergistic effects. Antibiotic resistance poses significant clinical challenges, leading to longer hospital stays, increased medical costs, and higher mortality rates. As resistant strains proliferate, the effectiveness of standard treatments diminishes, necessitating the use of more potent or toxic alternatives. This underscores the importance of careful antibiotic selection and combination strategies in clinical settings. The selection of antibacterial combinations should be strategic, aiming to maximize potential synergy while avoiding antagonistic interactions. Research using disc diffusion methods has shown that a co-spray-dried combination of ciprofloxacin and doxycycline hydrochloride in a 1:1 ratio resulted in negligible changes in inhibitory zone diameters against *Staphylococcus aureus*, *Pseudomonas aeruginosa*, and *Streptococcus pyogenes*. However, this combination has demonstrated some suppressive effects against* Escherichia coli*. Therefore, selecting effective antibiotic combinations requires a thorough understanding of both the measurement techniques and the target microorganisms [14].

Compounds of liquid formulation: For example, in both in vitro and in vivo models of *S. aureus* and *P. aeruginosa*, the combination of tobramycin with fosfomycin exhibited synergistic antibacterial activity. Fosfomycin enhanced the absorption of tobramycin in *P. aeruginosa* in a dose-dependent manner. It has been demonstrated that the co-administration of tobramycin with clarithromycin and colistin improves anti-biofilm activity. In phase II clinical studies, nebulized tobramycin combined with fosfomycin showed clinical antimicrobial effectiveness against *P. aeruginosa *in cystic fibrosis patients [15].

The use of the powdered substance: Using spray drying, Lee and colleagues developed binary combinations of ciprofloxacin and gemifloxacin hydrochloride salts, as well as ternary powders (binary plus lysozyme). The binary formulation demonstrated a synergistic antimicrobial effect against *P. aeruginosa*, according to a time-kill model. However, the ternary mixture showed no effect on *P. aeruginosa*, *S. aureus*, *K. pneumoniae*, or *Acinetobacter baumannii*. The ternary mixture's potential for strong mucolytic effects in cystic fibrosis patients warrants further investigation. The spray drying technique was also applied to combine tobramycin with other antibiotics, such as azithromycin, ceftazidime, or clarithromycin, to create powder formulations. Tobramycin and ceftazidime powders were spray-dried with dipalmitoyl phosphatidylcholine, albumin, and lactose [16]. 

Types of Formulation-Based Microbes

Antibacterial compositions for inhalation: In the United States, nebulization has thus far been approved only for tobramycin and aztreonam solutions. However, some European countries have authorized the nebulization of colistin methanesulfonate solution (also known as colistimethate sodium, CMS). Clinical studies are also underway for other antibiotics, including amikacin, ciprofloxacin, and levofloxacin. The only approved dry powder formulations in the USA and Europe are tobramycin (TOBI® Podhaler®) and colistin methanesulfonate (Colobreathe®). Vancomycin and ciprofloxacin dry powder formulations are nearing completion [17].

Formulations for bacteriophage: As an alternative to conventional antibiotics, phage therapy represents a promising approach to treating infections. Bacteriophages are viruses that infect and replicate within bacterial cells, with the potential to overcome protective barriers like biofilms that often shield bacteria from antibiotics. Phages can penetrate biofilms, reproduce locally to destroy bacteria, and enhance antibiotic effectiveness. To improve patient compliance and product stability, bacteriophages are being researched as dry powders suitable for inhalation. The physiological stability of phage powders is crucial, as it affects both the cost and efficacy of treatments. However, powder processing methods such as mechanical milling can damage sensitive biopharmaceuticals like proteins. 

Antiviral Compositions for Inhalation

Zanamivir: Zanamivir is approved for inhalation use in the US. The Relenza® Rotadisk® formulation combines 5 mg of zanamivir with 20 mg of lactose (GlaxoSmithKline). The recommended dosage involves inhaling two blisters twice daily for five days. It has been observed that influenza-infected patients aged five to 12 years experienced a significant reduction in the median duration of symptoms by 1.25 days. However, it is important to note that this dosing regimen may vary based on specific clinical guidelines or individual patient conditions. The inhaler has a low resistance of 0.022 kPa^1/2^/(l/min) and a flow rate of 90 l/min. At this flow rate, the Next Generation Impactor indicated a fine particle fraction (FPF) of less than 30%. FPF is a critical parameter in determining therapeutic efficacy, as it represents the proportion of aerosol particles that are small enough to reach the deep lung regions where effective drug delivery occurs. Although the pre-separator and device exhibited high powder retention rates of 45.45% and 20.9% (with an additional 5.2% noted), a low FPF can limit the actual amount of medication available for absorption in the lungs. By contrast, comparable lung dosage was achieved using an airway simulator that generated an inspiratory peak flow of 84 l/min, and 2.78 l/min using a physical airway model rather than a USP throat. Furthermore, atomic-force microscopy revealed that the adhesion force between spray-dried lactose (d_v, 0.5 = 150 µm with a geometric standard deviation of 1.8) and micronized zanamivir had quadrupled, which may affect the release and deposition of the drug in the lungs [18].

Laninamivir: A novel neuraminidase inhibitor, Laninamivir octanoate, is currently approved only in Japan (Inavir® by Daiichi Sankyo Company Ltd., Tokyo, Japan; and Biota Pharmaceuticals, Alpharetta, USA). Due to its long-lasting retention in the lungs, it requires only a single inhalation, compared to zanamivir's twice-daily regimen over a five-day treatment cycle. In animal studies, Laninamivir's half-life (t1/2) was found to be 41.4 hours, indicating the time required to reach 50% of its concentration following administration. However, it is important to note that pharmacokinetic (PK) values from animal studies may differ in humans. While lung levels remained above the IC50 (50% inhibitory concentration) even after 120 hours, patients with both type A and B influenza typically need a minimum of four days, excluding the days of administration, to recover from fever and flu symptoms.

Anti-influenza: Anti-flu medications are continually being developed. As new strains evolve, antiviral drugs targeting specific viral genes or proteins sensitive to mutation may become ineffective. Broad-spectrum antiviral drugs target common functions by various viruses rather than specific viral proteins, allowing them to remain effective even when the virus undergoes genetic changes. For instance, favipiravir inhibits viral RNA polymerase, which is crucial for replication, making it effective against multiple RNA viruses like influenza and Ebola. Remdesivir, which also targets viral polymerase, has shown effectiveness against a range of RNA viruses. In addition, ribavirin interferes with viral RNA synthesis and is used to treat hepatitis C and respiratory syncytial virus (RSV). By attacking key processes necessary for viral replication, these broad-spectrum antivirals can effectively combat viral infections, even as viruses mutate [19]. DAS181, ribavirin, recombinant human catalase, and Cidovir are among these medications. Table 1 lists the formulations of antiviral drugs [18,19].

**Table 1:** Antiviral drug formulation DAS 181: dendrimeric sialidase, FPF: fine particulate matter, H3N2: hemagglutinin type 3 and neuraminidase type 2, H1N1: hemagglutinin type 1 and neuraminidase type 1, N/A: not applicable

Active pharmaceutical ingredient	Targeting virus	Formulation	Delivery technique	Deposited lung dose	Dosing interval	Reference
Fludase® (a recombinant protein Inhaled anti-viral drugs that showed efficacy against different types of viruses. Binant protein with sialidase activity)	Human parainfluenza virus 3	DAS181 1mg/ml water	Used to nebulize AerogenProX	FPF (15μm) = 68.2%	Once daily for five days. Nebulization time: 8 min (For infant 18 months old)	[18]
		A stock solution of DAS181 10mg/ml water is diluted in saline to give1.3mg/ml Composition of dry powder is not specified.	Aerolizer®	Total DAS181 deposited 6.1mg FPF(1-5μm) = 68.2%	Once daily for five days. Nebulization time: 4 min (1st day),7 min (2nd day),7-14 min (3rd and 5th days) (females: 40-year-old)	[18]
	H3N2 inﬂuenzavirus	Ribavirin 100mg/ml in water	Nebulized with Aerotech II (CIS-USA, Bedford, MA)	Ribavirin 20.4 mg/kg body weight N/A	One capsule for five days. 10 mg DAS1881 in each capsule once daily for four days	[18]
Ribavirin used at a high dosage to expedite therapy	H1N1 inﬂuenzavirus	Recombinant human catalase in saline (33,000 U/ml).	Nebulized (The nebulizer's kind is unknown)	Sometimes N/A	Nebulization time: 30 min Two time a day for four days dose 1.5 ml/kg Once a day for three days. Three different doses: 0.5, 1.0, and 1.75 mg/kg.	[19]
	Micronized Cidovir	Rotating brush powder generator (Palas, Karlsruhe, Germany)			(Rabbit model)	[19]

Antifungal Formulations for Inhalation

Amphotericin: Adenosine inhibitors are used to treat life-threatening pulmonary fungal infections, with amphotericin B being the most widely studied and utilized antifungal drug for inhalation delivery. Amphotericin B is a membrane-active polyene macrolide with antifungal properties. Shah and Misra developed a dry powder form of liposomal amphotericin B. Most trials, however, have focused on evaluating the efficacy of nebulizing existing formulations, such as lipid-based formulations like Fungizone® (a deoxycholate formulation), Ambisome® (liposome-based), Abelect®, and Amphotec®.

Voriconazole: Voriconazole is a broad-spectrum antifungal medication available in both oral and intravenous forms (Vfend® and Captisol®). While effective, the limited water solubility of voriconazole (0.61 mg/ml at pH 7) presents challenges for formulation. Following the discontinuation of systemic voriconazole treatments due to significant side effects, inhaled voriconazole therapy demonstrated efficacy in three cases of severe aspergillosis. When delivered via inhalation, the patient's breathing pattern becomes crucial, as proper inhalation techniques, including slow and deep breaths, are essential for ensuring that the medication reaches the lower respiratory tract where it can effectively treat fungal infections. Rapid or shallow breathing can reduce the deposition of voriconazole in the lungs, leading to suboptimal therapeutic outcomes [20]. Without attempting to regulate the patient's breathing pattern, a 40 mg dose of voriconazole was nebulized directly into their lungs. Table 2 lists the formulations of antifungal drugs [20].

**Table 2:** Antifungal drug formulation PVPK30: polyvinylpyrrolidone K30

Drug	Formulation	Production method	Excipient	Fine particle fraction	Device	Test flow rate (1/Min)	Reference
Amphotericin B	Liposomal formulation for Nebulization	Commercially available AmBisome® for injection	Hydrogenated soya phosphatidylcholine (48%), Cholesterol (24%), Di stearoyl phosphatidyl glycerol (19%)	18.5%	Atomisor® NL9MP	15	[20]
	Suspension for Nebulization	Chitosan-stearic acid conjugate nano micelles by solvent evaporation	Chitosan-stearic acid with 17 or 60% substitution degree (60-95% w/w)	40-52%	Air-Jet nebulizer	60	[20]
	Suspension for Nebulisation	Non-ionic surfactant vesicles	Amphotericin B (100:1 w/w), lipids including Mono-n-hexa decyl ether tetra ethylene glycol, Cholesterol, and diacetyl phosphate (30 and 150 mM) Mannitol (30%), leucine (12%)	4-21%	Buxco® nebulization system	60	[20]
Voriconazole	Dry powder by spray drying micro-nano suspension	Crystalline microparticles in the absence of excipients and Amorphous nano-structured aggregated in the presence of excipients by thin film freezing	Phospholipid (0.36-3.47%), PVPK 30 (25-33%)	5-43%	HandiHaler®	60	[20]

Limitations

Despite the advancements in pulmonary drug delivery systems, there are several limitations associated with these technologies. For instance, the effectiveness of liposomal drug formulations can be hindered by issues such as drug leakage from the liposomes. Similarly, while nanoparticles offer controlled release and enhanced bioavailability, they may still face challenges related to production costs and potential toxicity. DPIs, particularly passive devices, depend heavily on the patient’s inhalation effort, which can be a disadvantage for those with reduced pulmonary function. In addition, nebulizers, especially jet types, often have drawbacks like long treatment times and bulkiness, while vibrating mesh nebulizers, despite their efficiency, are cost-prohibitive. Lastly, the formulation stability of dry powders in DPIs can be compromised under high humidity, and certain combinations of antibiotics in powder form may not exhibit the expected synergistic effects, posing further challenges in their clinical application.

## Conclusions

Understanding breakthroughs in cellular and molecular mechanisms and physiology is crucial for elucidating the molecular features and biochemical processes of the pulmonary system. Research in medical science enables a focus on the molecular mechanisms of diseases and the limitations of medication delivery systems. A good understanding of therapeutic drugs on how they work at the cellular and molecular levels, drug delivery systems, aerosol administration methods, pulmonary deposition patterns, and the different drug delivery devices is important for an effective pulmonary drug delivery system. The lungs are a vital organ, and a deeper understanding of their function will aid in developing effective medication delivery systems for chronic pulmonary disorders. Consequently, understanding these pathways is critical for maximizing the benefits and efficiency of existing pulmonary drug delivery methods.

Pharmaceutical chemists face numerous challenges and complexities in developing pulmonary medication delivery systems. Recent advancements in pulmonary drug delivery have transformed therapeutic approaches for respiratory diseases. Innovations such as smart inhalers, which track medication usage and improve adherence, and nanoparticle-based systems enhance drug stability and targeted delivery. In addition, microneedle arrays and lipid-based formulations facilitate efficient drug absorption and controlled release. The emergence of personalized inhalation devices through 3D printing allows for tailored treatments, while the development of inhalable biologics and gene therapies offers new avenues for targeting inflammatory lung conditions.

Addressing these challenges helps pharmaceutical scientists overcome technical and clinical obstacles in creating pulmonary medicines. In developing pulmonary drug delivery systems, regulatory guidelines ensure that inhalers and medications are safe, effective, and high-quality. Agencies like the FDA and EMA require complete testing of these products. A focus on patient-centric approaches also means that inhalers are designed to be easy to use and meet patient needs. By involving patients and healthcare providers in the development process, the goal is to create treatments that improve how well patients stick to their therapy and manage their respiratory conditions. Researchers have a wide range of options for in-vivo, ex-vivo, and in-vitro experiments to monitor drug absorption in animal models, facilitating the development of advanced strategies for pulmonary drug delivery.
